# Mesenteric Thrombosis

**Published:** 1914-04

**Authors:** 


MESENTERIC THROMBOSIS. Drouin and A Parcelier, *Jour. de Med. de Bordeaux,* August 17, 1913, reports a case in a man of 66, beginning with sudden severe circum-umbilical pain. Operation that evening, revealed 200-300 c.c. of sanguinolent serum in the peritoneum, with some clots, about one meter of small intestine being sharply distinguished by its dark color from the normal bowel. On account of the bad state of the patient, radical operation was abandoned. Necropsy verified the observation at operation, the necrotic portion beginning 2·20 meters from the pylorus.

